# Spontaneous Isolated Superior Mesenteric Artery Dissection Presenting as Chronic Postprandial Abdominal Pain: Conservative Management of a Yun Type IIb Case

**DOI:** 10.7759/cureus.108347

**Published:** 2026-05-06

**Authors:** Sujan Bohara, Amar P Rana, Adarsha Mahaseth, Mamata Shrestha, Amrit K C, Abhinandan Shrestha

**Affiliations:** 1 Internal Medicine, Dr. Iwamura Memorial Hospital, Bhaktapur, NPL; 2 General Medicine, Manipal College of Medical Sciences, Pokhara, NPL; 3 Medicine, Nepalese Army Institute of Health Sciences, Kathmandu, NPL; 4 Medicine, Nepalese Army Institute of Health sciences, Kathmandu, NPL; 5 General Medicine, Dr. Iwamura Memorial Hospital, Bhaktapur, NPL

**Keywords:** conservative management, ct angiography, false lumen thrombosis, intimal flap, isolated sma dissection, mesenteric artery dissection, mesenteric ischemia, postprandial abdominal pain, vascular imaging, yun classification

## Abstract

Spontaneous isolated superior mesenteric artery dissection (SISMAD) is an uncommon but increasingly recognized vascular cause of abdominal pain due to the widespread use of advanced imaging. Its clinical presentation is variable, ranging from incidental findings to acute or chronic abdominal pain, and optimal management remains individualized based on clinical stability and radiologic features.

We report the case of a 52-year-old male who presented with a six-month history of intermittent postprandial epigastric pain. Initial clinical and laboratory evaluations were unremarkable. Contrast-enhanced computed tomography aortography revealed a focal fusiform dilatation of the proximal superior mesenteric artery with a distinct intimal flap, resulting in separation into true and false lumens. The false lumen demonstrated partial thrombosis, while the true lumen appeared moderately narrowed, with an estimated luminal compromise of approximately 40-50% relative to the overall vessel diameter, but remained patent with preserved distal perfusion. There was no evidence of bowel ischemia or arterial rupture. Based on these findings, the diagnosis of isolated spontaneous superior mesenteric artery dissection, consistent with Yun type IIb morphology, was established.

Given the patient’s hemodynamic stability, absence of ischemic complications, and preserved mesenteric perfusion, a conservative management approach was adopted. Treatment included antiplatelet therapy, beta-blockade, statin therapy, and dietary modification. The patient demonstrated progressive symptomatic improvement and remained clinically stable at the six-month clinical follow-up without recurrent symptoms or clinical evidence of bowel ischemia. Follow-up imaging was not performed; therefore, radiologic disease progression or vascular remodeling could not be objectively assessed.

This case highlights the importance of considering SISMAD in patients presenting with chronic postprandial abdominal pain, even in the absence of traditional vascular risk factors. Early use of computed tomography angiography is critical for accurate diagnosis and risk stratification. In carefully selected patients without bowel ischemia, conservative management can be safe and effective, provided that close clinical monitoring and follow-up are ensured.

## Introduction

Spontaneous isolated superior mesenteric artery dissection (SISMAD) is an uncommon vascular disorder and a rare cause of abdominal pain, with a historically reported incidence of approximately 0.06%, a figure largely derived from autopsy/post-mortem data and subsequently cited in retrospective clinical and imaging-based series [1-4]. Although traditionally considered rare, its recognition has increased substantially in the era of high-resolution contrast-enhanced computed tomography angiography (CTA), which enables earlier and more accurate identification of mesenteric arterial pathology. The clinical spectrum is broad, ranging from incidental asymptomatic lesions to acute abdominal pain, chronic postprandial pain, mesenteric ischemia, aneurysmal degeneration, or, rarely, rupture [1-5].

Most reported patients are middle-aged men, and commonly described associations include hypertension and smoking, although diabetes appears less frequent than in other vascular diseases [4,5]. The diagnosis rests primarily on CTA, which characteristically demonstrates an intimal flap, true and false lumens, false lumen thrombosis, true lumen narrowing, and the longitudinal extent of the dissection, while simultaneously allowing assessment for bowel ischemia, branch involvement, and other visceral arterial abnormalities [2,3,6,7].

Several morphologic classification systems have been proposed to guide prognostication and treatment. Among the most widely used classifications, the Yun classification stratifies lesions according to true lumen patency, false lumen flow, thrombosis, and re-entry, whereas CT-based morphologic schemes further describe the extent of dissection and distal branch involvement [6-8]. The Yun classification was emphasized in this report due to its clinical relevance and widespread use in guiding management decisions, particularly in distinguishing flow characteristics and thrombosis patterns that influence conservative versus interventional strategies. Despite growing recognition of SISMAD, the optimal treatment strategy remains incompletely standardized. Current evidence and contemporary vascular guidance generally support conservative therapy as first-line treatment in hemodynamically stable patients without bowel ischemia or arterial rupture, reserving endovascular or surgical intervention for those with persistent symptoms, worsening stenosis, progression on imaging, aneurysmal enlargement, or ischemic complications [1,5,9-14].

The present case is clinically instructive because it illustrates an unusual chronic presentation of SISMAD with postprandial epigastric pain, focal fusiform dilatation of the superior mesenteric artery (SMA), partial false lumen thrombosis, and true lumen narrowing, without radiologic evidence of bowel compromise. Its favorable response to conservative management emphasizes the importance of timely vascular imaging, accurate morphologic characterization, and appropriate patient selection for non-operative therapy [15-18].

## Case presentation

A 52-year-old male healthcare worker presented to the outpatient department with a six-month history of intermittent upper abdominal pain. The pain was insidious in onset and progressively persistent, described as a dull, burning sensation localized to the epigastric region. It was characteristically postprandial, occurring within 30-60 minutes after meals, and was notably exacerbated by the intake of fatty or oily foods. The intensity of pain was moderate and non-radiating, with partial and temporary relief following the use of over-the-counter analgesics and proton pump inhibitors. There were no associated symptoms of nausea, vomiting, hematemesis, melena, altered bowel habits, or anorexia. Additionally, the patient denied any constitutional symptoms such as fever, weight loss, or fatigue.

The patient had no known comorbidities, including hypertension, diabetes mellitus, dyslipidemia, or cardiovascular disease. He was a non-smoker and did not consume alcohol. His past medical history was significant for a hiatus hernia, which had been managed conservatively, and a surgically repaired ulnar nerve injury. He was not on any long-term medications and had no known drug allergies. There was no personal or family history suggestive of connective tissue disorders, vasculitis, thromboembolic disease, or vascular aneurysms.

On physical examination at presentation, the patient was hemodynamically stable, with vital parameters within normal limits. General examination revealed no pallor, icterus, cyanosis, clubbing, or lymphadenopathy. Abdominal examination demonstrated a soft, non-distended abdomen with mild tenderness localized to the epigastric region on deep palpation. There was no guarding, rigidity, rebound tenderness, or palpable abdominal masses. No abdominal bruits were auscultated. Bowel sounds were normal and symmetrical. Examination of other systems, including cardiovascular, respiratory, and neurological systems, was unremarkable.

Baseline laboratory investigations were within normal limits (Table 1), effectively excluding acute inflammatory, infective, or hepatopancreatobiliary etiologies.

**Table 1:** Baseline laboratory investigations at presentation AST, aspartate aminotransferase; ALT, alanine aminotransferase; SGOT, serum glutamate oxaloacetate transaminase; SGPT, serum glutamate pyruvate transaminase; CRP, C-reactive protein

Parameter	Result	Reference Range
Hemoglobin	13.8 g/dL	13.0–17.0 g/dL
Total leukocyte count	7,200/mm³	4,000–11,000/mm³
Platelet count	2.5 × 10⁵/mm³	1.5–4.5 × 10⁵/mm³
Serum lactate	1.2 mmol/L	0.5–2.0 mmol/L
Serum creatinine	0.9 mg/dL	0.6–1.3 mg/dL
Blood urea nitrogen	14 mg/dL	7–20 mg/dL
Total bilirubin	0.8 mg/dL	0.2–1.2 mg/dL
AST (SGOT)	28 U/L	10–40 U/L
ALT (SGPT)	32 U/L	7–56 U/L
Alkaline phosphatase	85 U/L	44–147 U/L
Serum amylase	72 U/L	30–110 U/L
Serum lipase	45 U/L	10–60 U/L
CRP	2 mg/L	<5 mg/L

Given the chronicity of symptoms, their postprandial nature, and suboptimal response to empirical therapy, further evaluation with contrast-enhanced computed tomography (CT) aortography was performed to assess for possible vascular causes of mesenteric ischemia.

**Figure 1:**
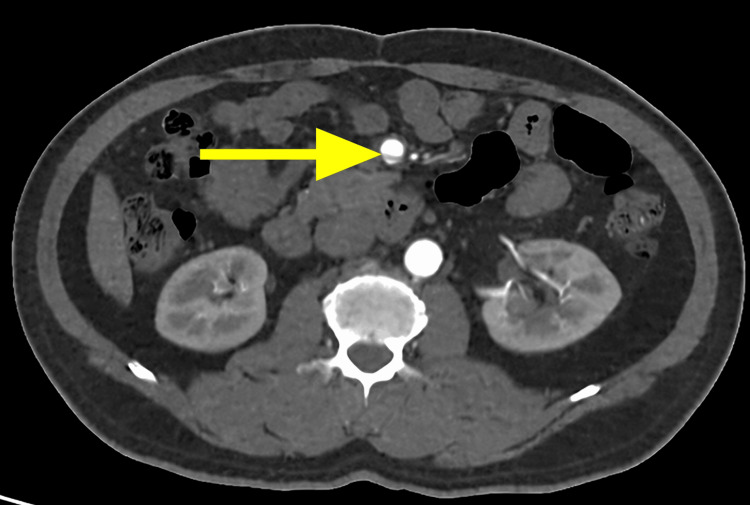
Axial contrast-enhanced CT aortography of the SMA. A linear intimal flap (arrow) is seen within the SMA, dividing the vessel into true and false luminal channels. CT, computed tomography; SMA, superior mesenteric artery

**Figure 2:**
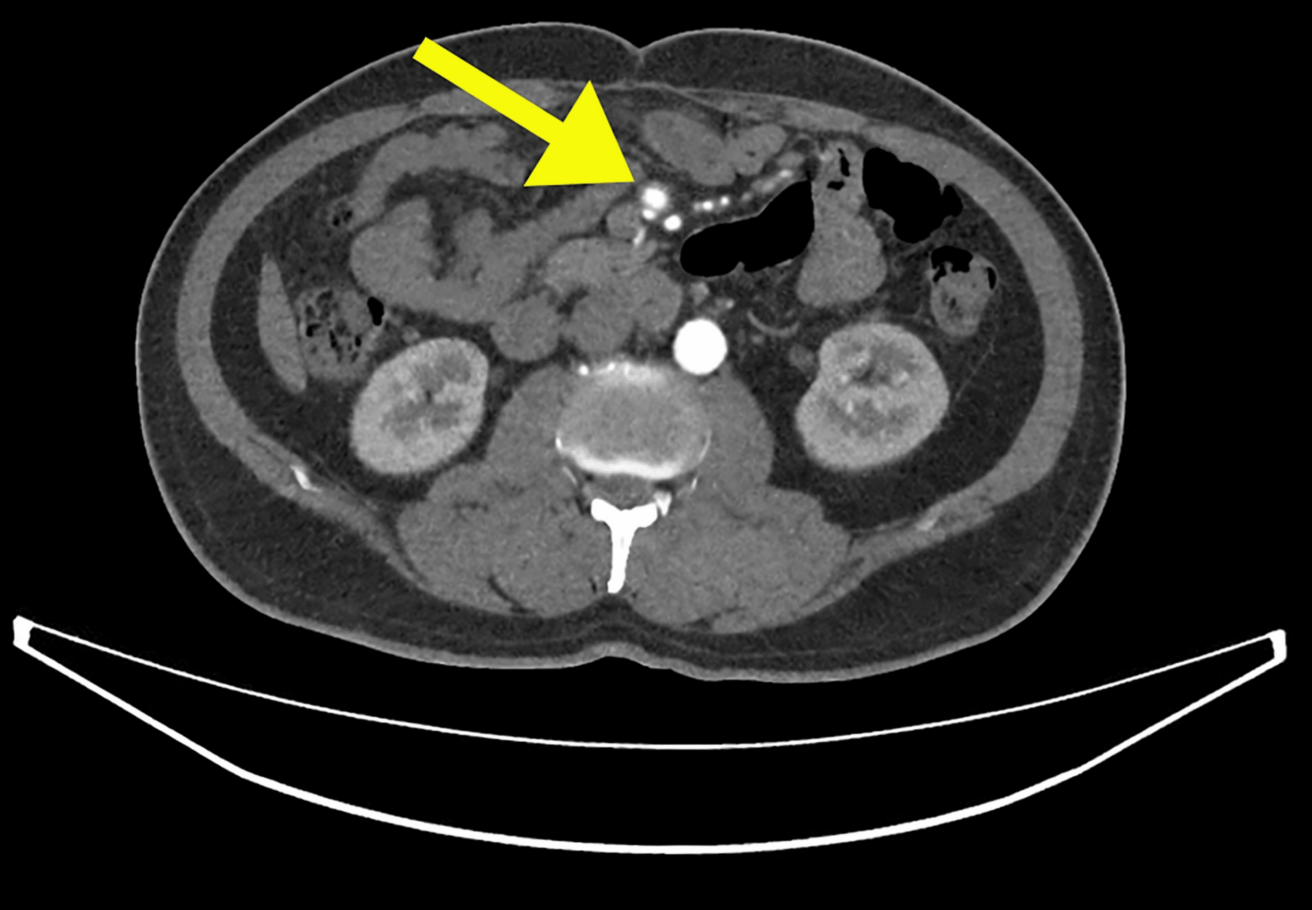
Axial CT aortography demonstrating superior mesenteric artery dissection. Partial thrombosis of the false lumen is seen (arrow), with moderate narrowing of the true lumen and preserved distal contrast opacification. CT, computed tomography

CT aortography revealed a focal fusiform dilatation of the proximal segment of the SMA, beginning approximately 8 mm distal to its origin from the abdominal aorta, with a maximum diameter of up to 11 mm. A distinct linear intimal flap was identified extending longitudinally over approximately 6.9 cm, resulting in division of the vessel lumen into true and false channels (Figures 1, 2). The false lumen was relatively larger and demonstrated partial thrombosis, more pronounced distally. The true lumen demonstrated moderate narrowing, with an estimated luminal compromise of approximately 40-50% relative to the overall vessel diameter, but remained patent with preserved antegrade contrast opacification extending into the distal SMA branches (Figures 1, 2).

**Figure 3:**
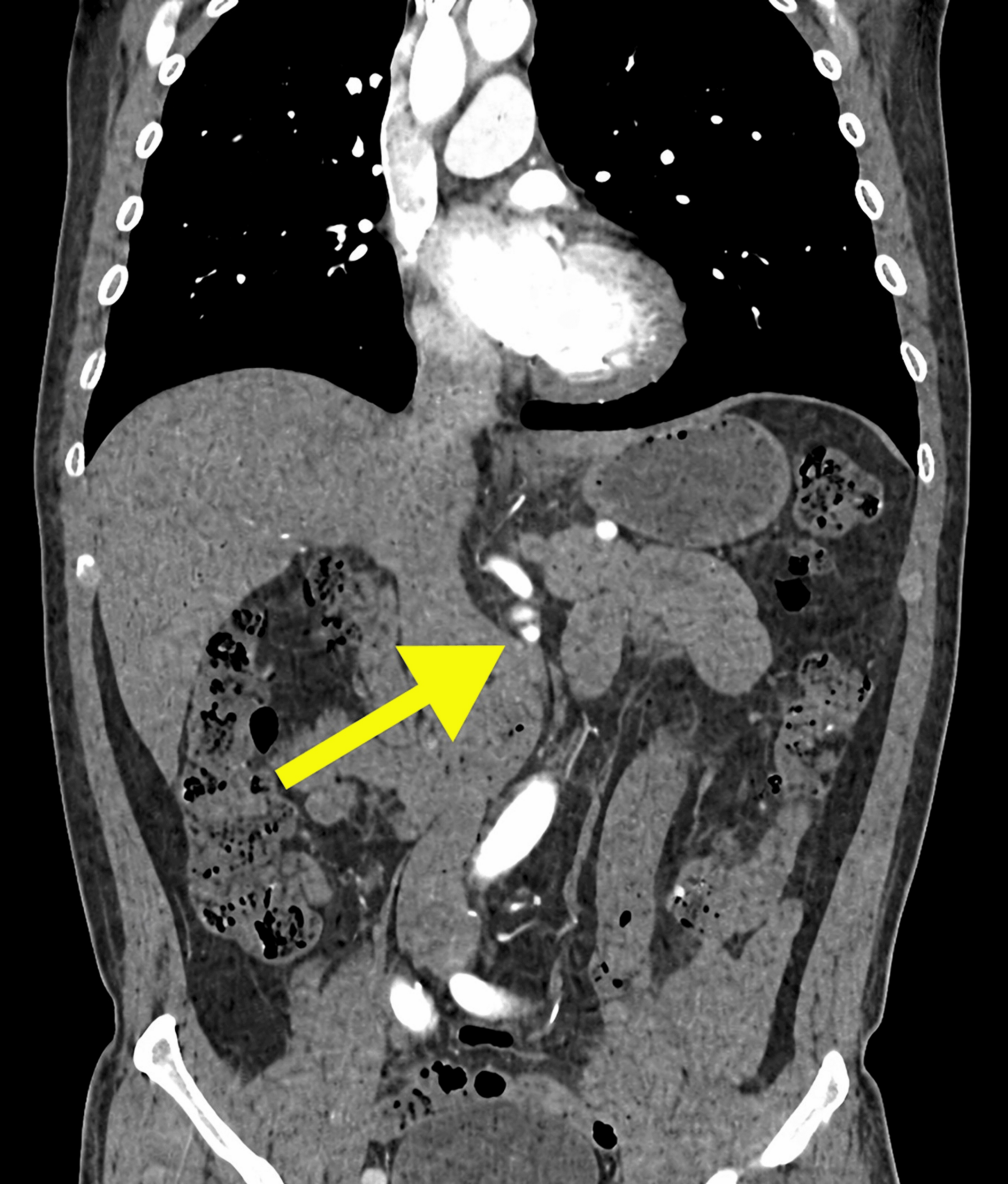
Coronal reformatted CT image of the SMA. A linear hypodense intimal flap (arrow) is visualized, clearly separating the true and false lumens. CT, computed tomography; SMA, superior mesenteric artery

**Figure 4:**
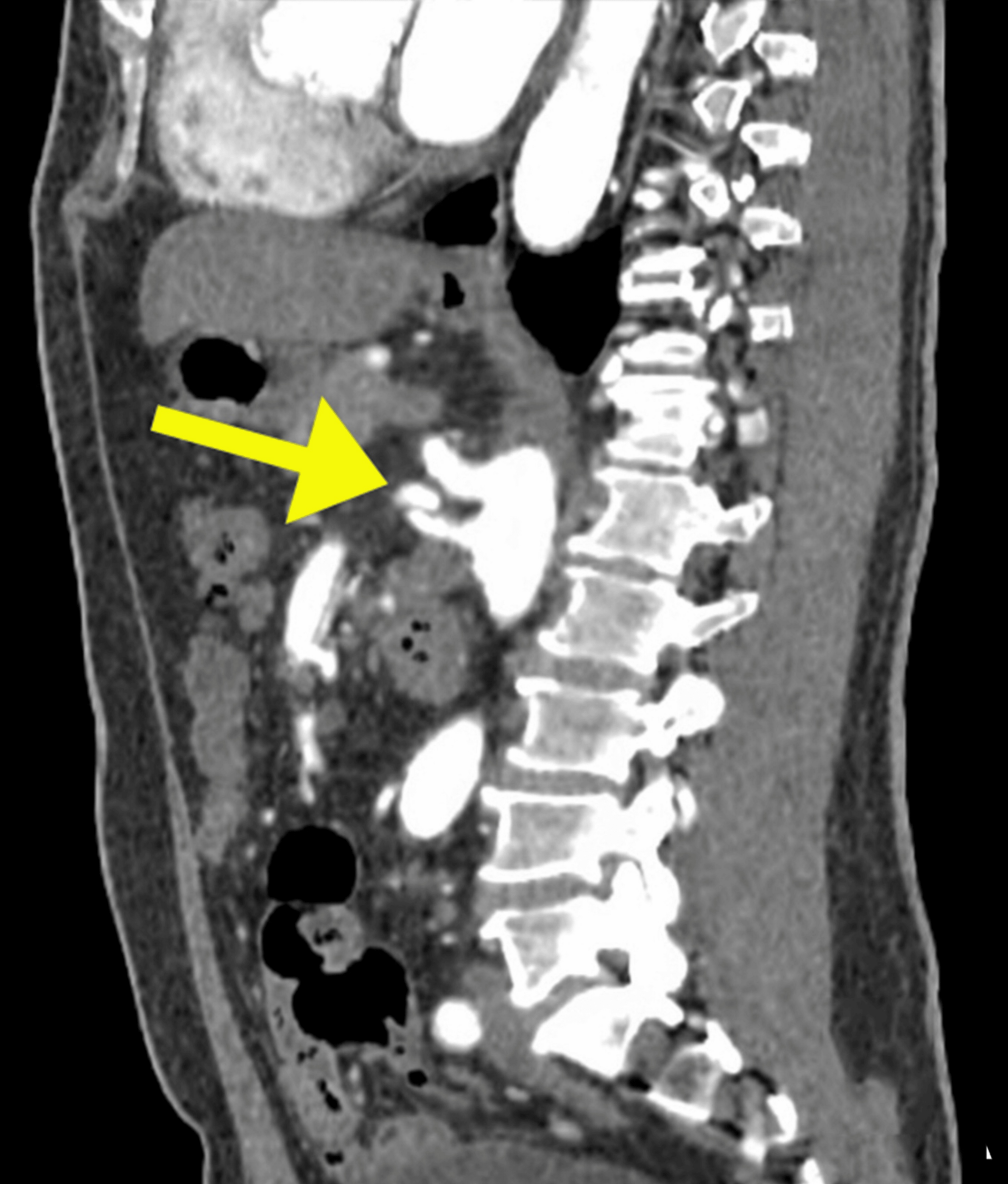
Sagittal reformatted CT image of the SMA. The dissecting intimal flap (arrow) demonstrates a dual-lumen configuration with partial thrombosis of the false lumen along the proximal SMA. CT, computed tomography; SMA, superior mesenteric artery

**Figure 5:**
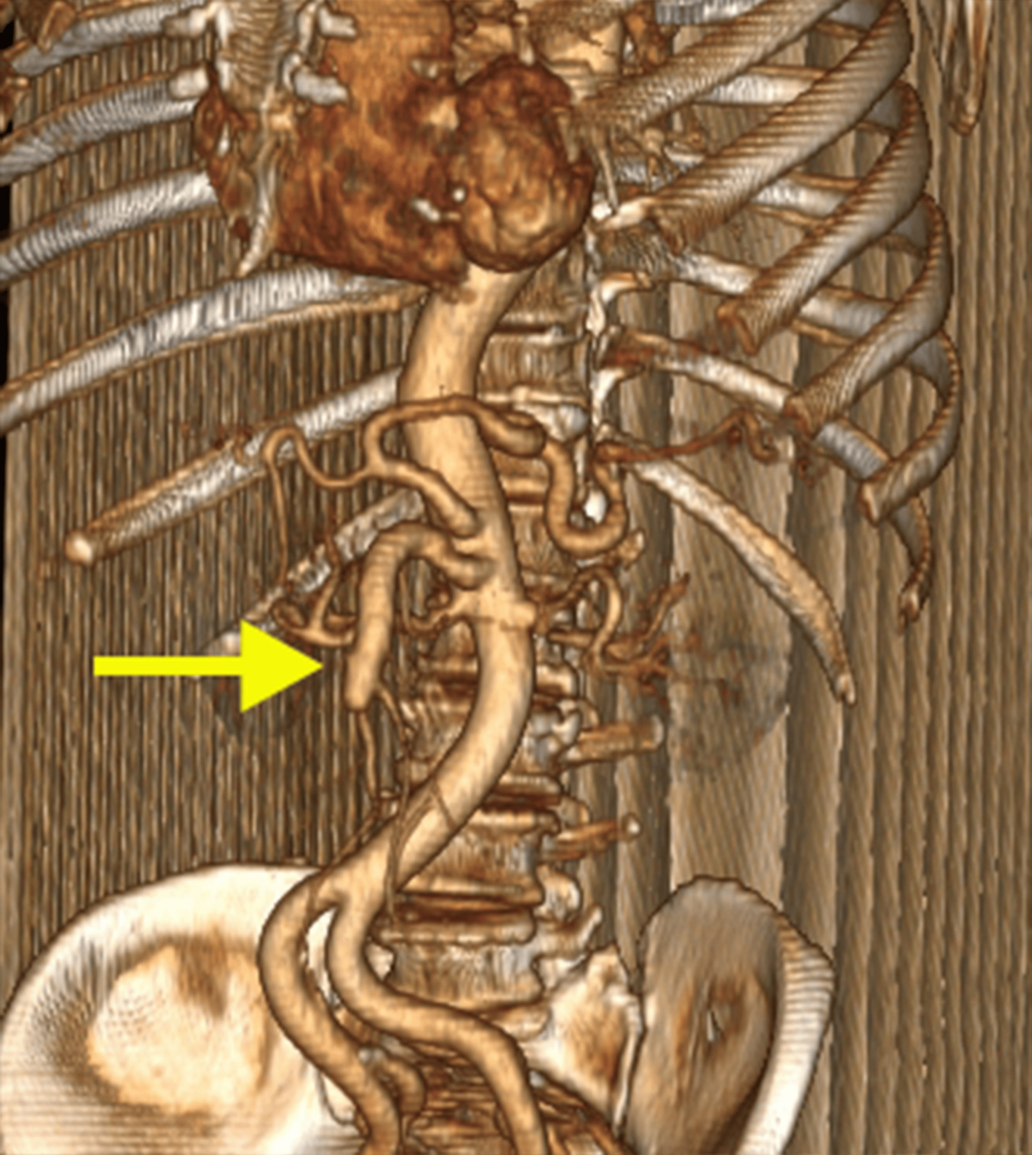
Three-dimensional volume-rendered CT angiography of the SMA. Focal fusiform dilatation of the proximal SMA is demonstrated; the arrow indicates the site of dissection distal to the arterial origin. CT, computed tomography; SMA, superior mesenteric artery

Axial CT images demonstrated the dissecting intimal flap as a low-attenuation linear structure within the SMA, clearly delineating the true and false lumens (Figure 1). The false lumen exhibited areas of hypoattenuation consistent with partial thrombosis, while the true lumen showed contrast enhancement, confirming maintained perfusion (Figure 2). Multiplanar reconstruction further enhanced visualization of the dissection. Coronal and sagittal reformatted images demonstrated the longitudinal extent of the intimal flap and the characteristic dual-lumen appearance of the vessel (Figures 3, 4). Three-dimensional volume-rendered CT angiography provided additional anatomical detail, illustrating the focal fusiform dilatation and tortuous course of the proximal SMA, with the site of dissection clearly identifiable (Figure 5). Because no prior vascular imaging was available, it could not be definitively determined whether the focal fusiform dilatation represented a pre-existing aneurysmal change or post-dissection expansion. Therefore, long-term imaging surveillance was advised to monitor for vascular remodeling, interval progression, or aneurysmal degeneration.

Importantly, there was no radiological evidence of bowel ischemia, such as bowel wall thickening, pneumatosis intestinalis, mesenteric fat stranding, or portal venous gas. The distal SMA branches were well opacified, indicating preserved mesenteric perfusion. Furthermore, there was no evidence of arterial rupture, perivascular hematoma, or extension of the dissection into the abdominal aorta or other major visceral arteries. The celiac trunk, renal arteries, inferior mesenteric artery, and iliac vessels appeared normal.

Collectively, these imaging findings were diagnostic of an SISMAD with preserved distal perfusion and no evidence of bowel compromise. Based on the morphological features, the dissection was classified as type IIb according to the Yun classification, characterized by a patent true lumen and a partially thrombosed false lumen without a re-entry site.

Given the absence of clinical or radiological features of mesenteric ischemia, preserved distal blood flow, and hemodynamic stability, a decision was made to pursue conservative management following multidisciplinary discussion involving gastroenterology and vascular surgery teams.

The patient was initiated on medical therapy, including aspirin 75 mg once daily, metoprolol 25 mg twice daily, and atorvastatin 20 mg once daily, along with dietary modifications emphasizing smaller, low-fat meals. He was closely monitored with regular clinical follow-up and blood pressure control.

Follow-up was scheduled at one month, three months, and six months, with clinical monitoring focused on recurrence or worsening of abdominal pain, features suggestive of bowel ischemia, and overall hemodynamic stability. Over subsequent follow-up visits, the patient demonstrated gradual and sustained symptomatic improvement, with marked reduction and eventual resolution of postprandial abdominal pain. At the six-month follow-up, he remained clinically stable without recurrent symptoms or clinical evidence of bowel ischemia. Follow-up imaging was not performed during this period; therefore, radiologic vascular remodeling, interval progression, or aneurysmal change could not be objectively assessed. Long-term imaging surveillance was advised to monitor for disease progression or aneurysmal degeneration.

## Discussion

This case highlights several important clinical and radiologic aspects of SISMAD. Although many patients present with acute abdominal pain, SISMAD may also manifest with subacute or chronic symptoms, particularly postprandial epigastric pain that mimics more common gastrointestinal conditions. Such presentations may delay diagnosis unless a vascular etiology is considered after routine laboratory evaluation and empiric gastrointestinal therapy prove unrevealing [2,3,17,18]. In the present case, the six-month history of meal-related pain, coupled with otherwise unremarkable laboratory findings and the absence of hepatopancreatobiliary pathology, made vascular imaging especially informative.

Given the chronic postprandial pain pattern, important differential diagnoses included chronic mesenteric ischemia secondary to atherosclerotic disease and median arcuate ligament syndrome. However, the absence of significant atherosclerotic narrowing, normal celiac axis morphology, and characteristic CTA findings of an intimal flap with a dual-lumen configuration supported the diagnosis of SISMAD.

The demographic profile of this patient is consistent with the existing literature, in which SISMAD predominantly affects men in the fifth to sixth decades of life [4,5]. In a systematic review and meta-analysis by Ullah et al., the mean age was 55.7 years, with approximately 80.6% of patients being male [4]. Our patient, a 52-year-old male, therefore fits the typical demographic pattern, although he lacked commonly reported vascular risk factors such as hypertension and smoking. This underscores that the absence of traditional cardiovascular risk factors does not exclude the diagnosis [5].

The imaging findings in this case were characteristic and diagnostically decisive. CTA demonstrated a proximal SMA dissection beginning shortly distal to the vessel origin, with a distinct intimal flap, separation into true and false luminal channels, partial thrombosis of the false lumen, moderate true lumen narrowing, and preserved distal perfusion without evidence of bowel ischemia. CTA remains the cornerstone of diagnosis, as it not only confirms the presence of dissection but also delineates its length, morphology, degree of stenosis, branch involvement, and associated complications [2,3,6,7]. In symptomatic patients, longer dissections, lesions located closer to the SMA origin, and greater degrees of true lumen compromise have been associated with increased symptom burden and potentially more complex management [6,7]. The location of the dissection shortly distal to the SMA origin may also be relevant from a pathophysiological standpoint. This proximal segment represents a transition zone between the relatively fixed arterial origin and the more mobile mesenteric portion of the vessel, where pulsatile flow, vessel curvature, and mechanical shear stress may contribute to intimal injury. In this patient, the dissection began approximately 8 mm distal to the SMA origin, making local hemodynamic stress a plausible contributing factor. Although the patient had a history of hiatus hernia, there was no clear anatomical or mechanistic evidence to suggest that it directly contributed to the SMA intimal tear. Therefore, the exact precipitating factor remains uncertain.

The morphologic pattern observed in this patient is best classified as Yun type IIb, characterized by a patent true lumen and a partially thrombosed false lumen without a re-entry site [6]. This subtype is clinically significant because thrombosis of the false lumen may compress the true lumen, resulting in fixed luminal narrowing while still permitting distal antegrade flow [6,11]. The Yun classification was emphasized in this report because it directly incorporates true lumen patency, false lumen thrombosis, and re-entry status, which are key features influencing conservative versus interventional management. In addition to the Yun classification, CT-based morphologic systems emphasize the importance of distal propagation and severity of true lumen stenosis in determining symptomatology and treatment response [7,8]. In this case, despite a relatively long dissected segment and true lumen narrowing, preserved distal perfusion and the absence of ischemic features supported an initial conservative approach.

From a pathophysiological perspective, the patient’s postprandial pain is plausibly explained by a demand-perfusion mismatch. Increased mesenteric blood flow requirements following meals may not be adequately met when the true lumen is narrowed by a thrombosed false lumen, resulting in relative hypoperfusion without progression to overt bowel infarction. This mechanism explains why some patients present with chronic or recurrent postprandial pain rather than acute catastrophic ischemia [6,7,17,18]. The absence of bowel wall thickening, pneumatosis intestinalis, mesenteric fat stranding, portal venous gas, or distal non-opacification in this case was therefore clinically reassuring, indicating hemodynamic compromise without transmural ischemia. Preserved distal perfusion despite true lumen narrowing may also be explained by compensatory collateral circulation, particularly through the pancreaticoduodenal arcades arising from the celiac arterial system, which may help maintain distal mesenteric flow despite partial compromise of the SMA lumen.

Management of SISMAD remains individualized; however, current evidence supports conservative therapy as the initial strategy in hemodynamically stable patients without peritonitis, bowel infarction, rupture, or progressive disease [1,5,9-14]. Conservative management typically includes bowel rest or dietary modification, analgesia, and strict blood pressure control [5,14]. The role of routine antithrombotic therapy remains controversial. Some studies, including those by Yun et al., have demonstrated no significant difference in outcomes between patients treated with or without anticoagulation or antiplatelet therapy, and systematic reviews have not shown consistent benefit for universal use [5,6]. Nonetheless, antiplatelet or anticoagulant therapy may be used selectively in cases with thrombosis, flow limitation, or perceived thromboembolic risk [16,17].

In this patient, conservative management with antiplatelet therapy, beta-blockade, statin therapy, and dietary modification resulted in clinical improvement without the need for intervention. Antiplatelet therapy was preferred over anticoagulation because the false lumen was already partially thrombosed, and there was no evidence of distal embolization, bowel ischemia, or an active thromboembolic process. The therapeutic goal was therefore to help maintain true lumen patency and reduce platelet-mediated thrombotic progression rather than to treat established embolic disease. Beta-blockade was employed to reduce arterial wall shear stress, including dP/dt-related stress on the intimal flap, thereby minimizing the theoretical risk of dissection propagation even in the absence of systemic hypertension. Statin therapy was used as part of vascular risk modification.

The favorable clinical course observed in this case is consistent with the natural history reported in larger observational studies. Park et al. demonstrated that most patients managed conservatively show either improvement or stability on follow-up imaging, with minimal radiologic progression [9]. Similarly, meta-analyses indicate that the majority of symptomatic patients treated conservatively achieve symptom resolution, with only a small proportion requiring delayed intervention [1,4,13]. Long-term imaging studies have further shown that vascular remodeling or stabilization is common, although complete normalization is not universal [19]. Therefore, while clinical improvement is reassuring, structured imaging follow-up remains essential. Early follow-up CTA is generally recommended at one to three months to monitor for progression, vascular remodeling, or aneurysmal degeneration, particularly in patients managed conservatively [9,19].

However, conservative management is not universally sufficient. Endovascular intervention plays a critical role in patients with persistent or worsening symptoms, significant luminal compromise, aneurysmal progression, or evidence of bowel ischemia [10-14,20]. Studies have demonstrated that endovascular therapy may achieve superior morphologic remodeling and reduce symptom recurrence in selected patients, although overall survival outcomes may be comparable when appropriate patient selection is applied [11,12]. The key clinical implication is that conservative management should be considered first-line in stable cases, but it must be accompanied by close monitoring and readiness to escalate treatment when indicated.

The contrast between this case and those requiring intervention is instructive. In previously reported cases, persistent abdominal pain with severe true lumen compromise necessitated endovascular stenting, resulting in rapid restoration of perfusion and symptom relief [20]. In contrast, our patient remained hemodynamically stable, had preserved distal perfusion, and showed clinical improvement with medical therapy alone over a six-month follow-up period. This reinforces a fundamental principle of SISMAD management: treatment decisions should be guided not solely by diagnosis but by a comprehensive assessment of symptoms, perfusion status, morphologic severity, and interval progression [10-14,20].

This case also aligns with emerging evidence from South Asian literature, where similar presentations have been reported in middle-aged men with abdominal pain, characteristic CTA findings, and favorable outcomes following conservative management [15,16]. However, this case adds clinical value due to its chronic presentation, long-segment dissection, partial false lumen thrombosis, true lumen narrowing, and associated focal fusiform dilatation. It demonstrates that anatomically significant disease may still follow a benign clinical course when perfusion is preserved and appropriate follow-up is maintained.

Overall, this case supports the growing consensus that SISMAD should be considered in the differential diagnosis of unexplained epigastric or postprandial abdominal pain, particularly in middle-aged men, even when routine laboratory findings are normal. Early use of CTA, careful morphologic classification, and appropriate risk stratification are essential for timely diagnosis and optimal management. Importantly, this case demonstrates that anatomically significant SISMAD does not necessarily mandate intervention if distal perfusion is preserved and that management decisions should be guided by clinical stability and perfusion status rather than imaging severity alone.

This report has several limitations. As a single-patient observation, it cannot determine comparative efficacy between conservative and endovascular treatment, nor can it establish causal relationships between specific components of medical therapy and the patient’s improvement. Although the imaging features were highly characteristic, no histopathologic confirmation was available, which is expected in successfully conservatively managed cases. In addition, the report is limited by the absence of advanced intravascular assessment, such as intravascular ultrasound, and by the lack of long-term serial CTA data demonstrating vascular remodeling over an extended follow-up period. Finally, because the patient had preserved distal flow and no bowel ischemia, the conclusions of this case are most applicable to stable, uncomplicated SISMAD and should not be generalized to patients presenting with peritonitis, progressive ischemia, rupture, or severe refractory symptoms.

## Conclusions

SISMAD is an uncommon but important vascular cause of abdominal pain that may present in a chronic postprandial pattern and closely mimic benign gastrointestinal disease. CTA is essential for diagnosis, morphologic classification, assessment of distal perfusion, and exclusion of bowel ischemia. This case of Yun type IIb SISMAD with false lumen thrombosis, true lumen narrowing, and preserved distal perfusion demonstrates that conservative treatment can be safe and effective in carefully selected, hemodynamically stable patients without ischemic complications. Close clinical follow-up and planned imaging surveillance remain crucial, as demonstrated by the favorable clinical outcome observed over a six-month follow-up period, while endovascular or surgical intervention should be reserved for persistent symptoms, progressive luminal compromise, aneurysmal change, or intestinal ischemia.

## References

[1] Karaolanis G, Antonopoulos C, Tsilimigras DI, Moris D, Moulakakis K (2019). Spontaneous isolated superior mesenteric artery dissection: Systematic review and meta-analysis. Vascular.

[2] Mei J, Jia Z (2023). Isolated superior mesenteric artery dissection: an updated review of the literature. J Interv Med.

[3] Gang C, Xiujuan G, Yingjiang X, Xun C, Dan S, Jianyong L, Bi J (2020). Accurate diagnosis and treatment of isolated mesenteric artery dissections. Vascular.

[4] Ullah W, Mukhtar M, Abdullah HM (2019). Diagnosis and management of isolated superior mesenteric artery dissection: a systematic review and meta-analysis. Korean Circ J.

[5] Acosta S, Gonçalves FB (2021). Management of spontaneous isolated mesenteric artery dissection: a systematic review. Scand J Surg.

[6] Yun WS, Kim YW, Park KB (2009). Clinical and angiographic follow-up of spontaneous isolated superior mesenteric artery dissection. Eur J Vasc Endovasc Surg.

[7] Luan JY, Li X (2013). Computed tomography imaging features and classification of isolated dissection of the superior mesenteric artery. Eur J Vasc Endovasc Surg.

[8] Li DL, He YY, Alkalei AM (2014). Management strategy for spontaneous isolated dissection of the superior mesenteric artery based on morphologic classification. J Vasc Surg.

[9] Park YJ, Park KB, Kim DI, Do YS, Kim DK, Kim YW (2011). Natural history of spontaneous isolated superior mesenteric artery dissection derived from follow-up after conservative treatment. J Vasc Surg.

[10] Chen X, Xu L, Xu Z (2022). Analysis of safety and efficacy of conservative treatment and endovascular treatment in patients with spontaneous isolated mesenteric artery dissection. Front Surg.

[11] Yu SH, Hii IH, Wu IH (2022). Comparison of superior mesenteric artery remodeling and clinical outcomes between conservative or endovascular treatment in spontaneous isolated superior mesenteric artery dissection. J Clin Med.

[12] Yang Y, Han T, Lin C (2025). Comparison of clinical outcomes between medical or/and endovascular therapy in spontaneous isolated superior mesenteric artery dissection patients without bowel ischemia. Asian J Surg.

[13] Kimura Y, Kato T, Inoko M (2018). Outcomes of treatment strategies for isolated spontaneous dissection of the superior mesenteric artery: a systematic review. Ann Vasc Surg.

[14] Koelemay MJ, Geelkerken RH, Kärkkäinen J (2025). Editor's Choice - European Society for Vascular Surgery (ESVS) 2025 Clinical Practice Guidelines on the Management of Diseases of the Mesenteric and Renal Arteries and Veins. Eur J Vasc Endovasc Surg.

[15] Anuroop J, Gandham S, Meraj MD, Moorthy NLN (2024). Uncommon abdominal pain: a case report on spontaneous superior mesenteric artery dissection. Indian J Case Rep.

[16] Perumal SR, Malathy K (2024). Isolated case of superior mesenteric artery dissection- a case report. J Clin Diagn Res.

[17] Daoud H, Abugroun A, Subahi A, Khalaf H (2018). Isolated superior mesenteric artery dissection: a case report and literature review. Gastroenterology Res.

[18] Yagnik V (2017). Isolated superior mesenteric artery dissection: a rare cause of chronic abdominal pain. Gastroenterol Hepatol Open Access.

[19] Jang JH, Cho BS, Ahn HY, Lee S, Kim H, Kim CN (2020). Optimal treatment strategy and natural history of isolated superior mesenteric artery dissection based on long-term follow-up CT findings. Ann Vasc Surg.

[20] Kim YJ, Beeman BR (2021). Symptomatic spontaneous superior mesenteric artery dissection treated with endovascular stent repair. J Surg Case Rep.

